# Acute Toxicity of the Antifouling Compound Butenolide in Non-Target Organisms

**DOI:** 10.1371/journal.pone.0023803

**Published:** 2011-08-29

**Authors:** Yi-Fan Zhang, Kang Xiao, Kondethimmanahalli H. Chandramouli, Ying Xu, Ke Pan, Wen-Xiong Wang, Pei-Yuan Qian

**Affiliations:** 1 KAUST Collaborative Research Program, Division of Life Science, Hong Kong University of Science and Technology, Hong Kong SAR, China; 2 Division of Life Science, Hong Kong University of Science and Technology, Hong Kong, SAR, China; University of Akron, United States of America

## Abstract

Butenolide [5-octylfuran-2(5H)-one] is a recently discovered and very promising anti-marine-fouling compound. In this study, the acute toxicity of butenolide was assessed in several non-target organisms, including micro algae, crustaceans, and fish. Results were compared with previously reported results on the effective concentrations used on fouling (target) organisms. According to OECD's guideline, the predicted no effect concentration (PNEC) was 0.168 µg l^−1^, which was among one of the highest in representative new biocides. Mechanistically, the phenotype of butenolide-treated *Danio rerio* (zebrafish) embryos was similar to the phenotype of the pro-caspase-3 over-expression mutant with pericardial edema, small eyes, small brains, and increased numbers of apoptotic cells in the bodies of zebrafish embryos. Butenolide also induced apoptosis in HeLa cells, with the activation of c-Jun N-terminal kinases (JNK), Bcl-2 family proteins, and caspases and proteasomes/lysosomes involved in this process. This is the first detailed toxicity and toxicology study on this antifouling compound.

## Introduction

**Figure 1:**
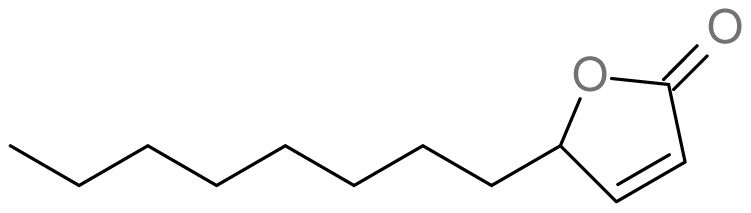
The chemical structure of butenolide [5-octylfuran-2(5H)-one].

Biofouling is one of the most serious problems in the maritime industry and aquaculture development. In the marine environment, submerged surfaces are often colonized by marine organisms that have come to be called biofoulers, which are marine organisms that attach to submerged surfaces. Biofoulers increase the weight, drag and surface corrosion of ships, and lead to huge costs to maintain mariculture systems and seawater pipelines [1]. Antifouling compounds are used as biocides in marine paints that are coated on the submerged surfaces to control the preponderance of biofoulers. It is estimated that without antifouling measures, the fuel consumption of ships would increase up to 40% [2]. However, the toxicity of antifouling compounds is a major concern. Tributyltin was a widely used antifouling compound, but it was completely banned in 2008 from the world's oceans because of concerns over its toxicity [3]. Several alternative antifouling compounds have replaced tributyltin, although most of them are still too toxic to be used for the long term [4]. Better and less toxic antifouling compounds are needed. A chemically synthesized butenolide, 5-octylfuran-2(5H)-one (Figure 1), is a very promising antifouling compound that has been recently designed and patented by our laboratory. This compound exhibits broad anti-fouling activity against major fouling species, such as barnacles, bryozoans and the tube-building polychaetes. Its antifouling activity has been demonstrated in a field test [5]. The preliminary toxicity study showed that it has very low toxicity in its target organisms as indicated by the high pharmaceutical ratios (LC_50_/EC_50_), and it has a simple chemical structure that makes it easy to be synthesized [5]. The toxicity of this butenolide in other non-target marine organisms has not yet been assessed in detail. For any new commercial antifouling compound, the predicted no effect concentration (PNEC) should be lower than the predicted environmental concentration (PEC) both inside harbors and in shipping lanes [6]. Besides determining its PNEC, it is also important to know the mechanism of toxicity of a compound. However, due to the paucity of molecular reporters, it is very difficult to study toxicology in marine organisms. We therefore used other model organisms to study the possible mode of action of butenolide. Zebrafish is widely used in pharmacology/toxicology studies, because it is small, optically transparent, accessible during development *ex utero*, and permeable to small molecules. Its embryogenesis is also very well characterized and a database of developmental defects (www.zfin.org) in zebrafish (*Danio rerio*) is available. Developmental defects in zebrafish caused by small molecules can be linked to a specific genetic pathway known to cause the same defect [7], [8]. On the other hand, cell cultures are more suitable for in-depth molecular mechanism studies, since they are simple and easy to control, and also have a large molecular toolbox available. In this study, the PNEC of butenolide was assessed using a toxicity study on non-target organisms and the previously reported toxicity data on fouling (target) organisms [5]. Then, the acute toxicology of butenolide was investigated both with zebrafish embryos and with the HeLa cell line.

## Materials and Methods

### Chemicals

The antifouling compound 5-octylfuran-2(5H)-one (here referred to as butenolide) was synthesized by Shanghai Medicilon Inc (Shanghai, China) The chemical structure for this compound is shown in Figure 1).

### Animal/cell culture and toxicity tests

Tables 1 and 2 summarize the methods used in the cell and animal culturing and toxicity tests, respectively. To identify a suitable cell line for this toxicology study, the dosage-dependent cytotoxicity of butenolide was determined in several cell lines with a MTT (3-(4,5-Dimethylthiazol-2-yl)-2,5-diphenyltetrazolium bromide) assay [9]. From HeLa, SF9 cells or primary neuron cultures, the supernatant was removed and 20 µl of MTT (2.5 mg ml^−1^) were added to each well. After incubation at 37°C for 4 h, 100 µl of dimethyl sulfoxide (DMSO) were added to each well and incubated for 20 min. The absorbance of each well was then measured at 570 nm by a Thermo scientific Multiskan® FC multiplate photometer (Waltham, MA, USA). The inhibitory effect or cytotoxicity of butenolide was then calculated based on the following equation:
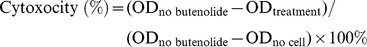
To HL-60 and K562 cells, 20 µl of MTT (2.5 mg ml^−1^) were added to each well and incubated for 4 h. They were centrifuged for 5 min at 1000 rpm and 100 µl of DMSO was added to the pellet and incubated for 20 min to solubilize the dark-blue formazan. Absorbance of the solution was measured at 570 nm and the cytotoxicity was calculated as described above.

**Table 1:** Source and culture methods of organisms used in the toxicity tests.

	Toxicity test preparation		
	Culture medium	Culture condition	Source of organism
HeLa cell	1	Seeded at 4000–5000 cells well^−1^, 24 h in 5% CO_2_ at 37°C	ATCC, Manassas, VA
Ptk2 cell	2	Seeded at 50000 cells well^−1^, 24 h in 5% CO_2_ at 37°C	ATCC, Manassas, VA
Sf9 cell	3	Seeded at 4000–5000 cells well^−1^, 24 h at 28°C, 75 rpm shaking	ATCC, Manassas, VA
HL-60 cell	4	Seeded at 50000 cells well^−1^, 24 h in 5% CO_2_ at 37°C	ATCC, Manassas, VA
K562 cell	4	Seeded at 50000 cells well^−1^, 24 h in 5% CO_2_ at 37°C	ATCC, Manassas, VA
Primary cortical neuron	12	Seeded at 6000 cells well^−1^, 15 d in 5% CO_2_ at 37°C. One third of the medium was replaced by fresh medium every 4 days of culture	Embryonic day 18 (E18) rats [35]
*Melita longidactyla*	5	Animals of 0.5–1 cm were maintained >24 h at 22±1°C	Hong Kong coastal waters
*Tigriopus japonicus*	5	Maintained >48 h under 22±1°C, fed with micro algae *Isochrysis galbana*	24.434335N,118.090925E
*Daphnia magna*	7	Fed stock daphnids with green alga *Chlamydomonas reinhardtii*. Collect young (<24 h) daphnids	Institute of Hydrobiology, Chinese Academy of Sciences
*Lutjanus erythropterus*	5	Juvenile fish of 3–4 cm were maintained >24 h at 22±1°C	Aqua farm in Shen Zhen, China
*Danio rerio*	10	Cultured at 28°C. Collect freshly fertilized embryos	Provided by Dr. Zi-Long Wen
*Skeletonema costatum*	11	Cultured at 24°C to exponential growth phase and seeded at 8*10^8^ cells ml^−1^	Hong Kong coastal waters

1—Minimum essential medium (MEM) containing 10% fetal bovine serum (FBS), 100 mg l^−1^ penicillin and 100 mg l^−1^ streptomycin.

2—Dulbecco medium supplemented with 10% heat-inactivated fetal calf serum.

3—Sf-900 II SFM.

4—RPMI-1640, supplemented with 10% heat-inactivated fetal calf serum.

5—Fully aerated seawater at salinity: 33±1‰.

6—Fully aerated 0.22 µm filtered seawater at salinity: 33±1‰.

7—Glass-fiber (GF/C Whatman, Maidstone, UK) filtered freshwater.

8—ISO testing water (1) [36].

9—Fully aerated 1 µm filtered seawater at salinity: 33±1‰.

10—60 µg ml^−1^ instant ocean sea salts dissolved in water.

11—f/2 culture medium [37].

12—Neural basal medium with B27 (Invitrogen, Carlsbad, CA, USA) and 0.5 mM GlutaMAX.

**Table 2:** Toxicity test methods.^1^

	Test solution	Tested butenolide concentrations (µg ml^−1^)	Toxicity test set up	Endpoint
HeLa cell	1	0, 5, 10, 25, 50, 75, 100, 200	96-well plate, 3 replicates	17 h cytotoxicity
Ptk2 cell	2	0, 6.25, 12.5, 25, 50, 75, 150	96-well plate, 3 replicates	12 h cytotoxicity
Sf9 cell	3	0, 5, 10, 25, 50, 75, 100, 200	96-well plate, 3 replicates	24 h cytotoxicity
HL-60 cell	4	0, 12.5, 25, 50, 75	96-well plate, 3 replicates	24 h cytotoxicity
K562 cell	4	0, 12.5, 25, 50, 75	96-well plate, 3 replicates	24 h cytotoxicity
Primary cortical neuron	12	0, 6.5, 12, 25, 50, 100	96-well plate, 3 replicates	48 h cytotoxicity
*Melita longidactyla*	6	0, 1, 1.75, 2.5, 3.25, 5	10 animals per 100 ml test solution×4 replicates	48 h lethality
*Tigriopus japonicus*	6	0, 0.5, 1, 1.75, 2.5, 3, 4	10 animals per 25 ml glass beaker containing 10 ml test solution×4 replicates	48 h lethality
*Daphnia magna*	8	0, 0.032, 0.16, 0.80, 4.0, 20	10 animals per 50 ml Falcon™ tube containing 25 ml test solution×4 replicates	48 h immobilisation
*Lutjanus erythropterus*	9	0, 0.06, 0.14, 0.29, 0.56, 0.70, 1.0, 1.5, 2.0, 2.5	10 fishes per 5 L flask containing 4 L test solution×3 replicates	48 h lethality
*Danio rerio*	10	0, 0.5, 1.0, 1.25, 2.5, 3.0, 3.5, 4.0, 4.5, 5.0	≤5 embros well^−1^ in 24-well plate (1 ml test solution well^−1^)	Development of pericardial edema and lethality at 55pfh (for EC_50_ and LC_50_, respectively)
*Skeletonema costatum*	11	0, 0.08, 0.4, 2, 10, 50	24-well plate, 1.35×10^16^ quanta sec^−1^ cm^−2^, 14 h/10 h light/dark cycle, 4 replicates	5 d IC_50_

1 Please see Table 1 for footnotes.

Cell cultures of *Skeletonema costatum*, in the exponential growth stage were diluted 20 fold in an f/2 culture medium (Table 2). The cell densities at the onset and completion of the experiment were measured by counting on a hemocytometer [10].

### Calculation of predicted no effect concentration (PNEC)

The predicted no effect concentration (PNEC) was calculated according to the equation below using the lowest no effect concentration (NEC) or L(E)C_50_ and assessment factor (AF) [4], [6].
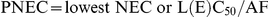



### Histological analysis and light microscopy

Zebrafish embryos treated with 1.25 µg ml^−1^ butenolide from the two-cell stage or the control embryos were fixed at 48 hours post fertilization (hpf) in 10% formalin in PBS overnight at 4°C. The samples were dehydrated in ethanol and infiltrated in paraffin resin (paraplast plus, McCormick Scientific, Richmond, USA) following the manufacturer's instructions. The specimens were then sectioned at 7 µm using a Leica Reichert-Jung 820-II Histocut Microtome (Leica Microsystems, Wetzlar, Germany). Histological hematoxylin-eosin (HE) staining of the sections was subsequently carried out using standard protocols [11]. The sections were then examined under an Olympus BX51 upright microscope connected to a Spot insight QE digital camera. The whole mount/live zebrafish embryos were manually dechorionated and examined under a Nikon MULTIZOOM AZ100 fluorescent microscope (NIKON CORPORATION, Tokyo, Japan) connected to a SPOT® FLEX color camera (SPOT Imaging Solutions, Diagnostic Instruments, Inc., Sterling Heights, MI, USA). Images were taken with the SPOT® advanced software (SPOT Imaging Solutions, Diagnostic Instruments, Inc., Sterling Heights, MI, USA) and then analyzed with Helicon focus (Helicon Soft Ltd., Kharkov, Ukraine) (for whole mount images) and Adobe Photoshop CS3 (Adobe Systems Incorporated, San Jose, CA, USA).

### Terminal deoxynucleotidyl transferase dUTP nick end labeling (TUNEL) assay

Zebrafish embryos treated with 1.25 µg ml^−1^ butenolide from the two-cell stage or the control embryos were fixed at 24 hpf in 4% paraformaldehyde in PBS for 45 min at room temperature. Whole-mount TUNEL staining of zebrafish embryos was performed using a fluorescein *in situ* cell death detection kit (Roche Applied Science, Indianapolis, USA) according to the manufacturer's protocol. Images were taken under a Nikon MULTIZOOM AZ100 fluorescent microscope with a GFP-B filter set (EX 460–500 nm, DM 505 nm, BA 510–560 nm) and a SPOT® FLEX color camera as described above.

### Western blotting

HeLa cells were cultured in 60 mm Petri-dishes. Cells at different time points after butenolide treatment were collected and lysed in NP-40 lysis buffer (50 mM Tris-HCl, pH 8.0, 150 mM NaCl, and 1% NP-40) in the presence of protease inhibitors. Whole cell lysates (60 µg lane^−1^) were separated on 10% SDS-PAGE and transferred onto a Hybond ECL nitrocellulose membrane (Amersham Biosciences, Piscataway, NJ, USA). After blocking, the membranes were incubated for 3 h at room temperature or overnight at 4°C with antibodies at a dilution of 1∶1000. The membranes were then incubated with horseradish peroxidase-conjugated secondary antibody at a dilution of 1∶5000 for 1 h and developed using Immobilon™ Western detection reagents (Millipore, Billerica, MA, USA).Information on the antibodies used in the experiments is summarized in Table 3.

**Table 3:** Antibodies used in this study.

Antigen	Antibody	Source
Mcl-1	Rabbit anti-Mcl-1 (S-19) polyclonal antibody	Santa CruZ Biotechnology, Inc. (Santa Cruz, CA, USA)
Bax	Rabbit anti-Bax polyclonal antibody	Cell Signaling Technology, Inc. (Danvers, MA, USA)
PUMA	Rabbit anti-PUMA polyclonal antibody	Cell Signaling Technology, Inc. (Danvers, MA, USA)
Phospho-JNK	Rabbit anti-phospho-SAPK/JNK (Thr183/Tyr185) polyclonal antibody	Cell Signaling Technology, Inc. (Danvers, MA, USA)
Phospho-p38	Rabbit anti-phospho-p38 MAPK (Thr180/Tyr182) polyclonal antibody	Cell Signaling Technology, Inc. (Danvers, MA, USA)
Phospho-ERK	Rabbit anti-phospho-p44/42 MAPK (Erk1/2) (Thr202/Tyr204) polyclonal antibody	Cell Signaling Technology, Inc. (Danvers, MA, USA)
PARP	Mouse anti-PARP (F-2) monoclonal antibody	Santa CruZ Biotechnology, Inc. (Santa Cruz, CA, USA)
GADPH	Rabbit GAPDH (14C10) monoclonal Antibody	Cell Signaling Technology, Inc. (Danvers, MA, USA)
Cdc2	mouse anti-cdc2 p34 (17) monoclonal antibody	Santa CruZ Biotechnology, Inc. (Santa Cruz, CA, USA)
Rabbit IgG	Goat anti rabbit IgG-peroxidase antibody	Sigma-Aldrich (St. Louis, Missouri, USA)
Mouse IgG	anti-mouse IgG, HRP-linked antibody	Cell Signaling Technology, Inc. (Danvers, MA, USA)

### Apoptosis suppression assay

To test the inhibitors of the apoptosis regulators, HeLa cells were seeded into a 24-well culture plate. Inhibitors were added 1 h before the application of 100 µg ml^−1^ butenolide. The inhibitors tested were 10 µM ALLN (N-Acetyl-Leu-Leu-Nle-CHO), 10 µM MG132 (Calbiochem, La Jolla, CA, USA), 50 µM Z-VAD-FMK (Promega, Madison, WI, USA), and 50 µM SP600125 (LC Laboratory, Woburn, MA, USA). The cytotoxicity was measured at 4 h by the MTT assay as described above. To investigate the effects of over-expression of Bcl-2 family proteins on butenolide-induced apoptosis, HeLa cells seeded in 35 mm Petri dishes were transfected with 1 µg of empty vector, Bcl-2, Mcl-1 or Bcl-XL constructs by lipofectamine 2000 (Invitrogen, Carlsbad, CA, USA) and incubated in an MEM medium containing 5% FBS at 37°C for 24 h before the application of 100 µg ml^−1^ of butenolide. The Bcl-2, Mcl-1 and Bcl-XL constructs tagged with green fluorescent protein were generously provided by Professor Donald C. Chang of HKUST. The cell toxicity was measured after 3 h of butenolide treatment by counting dead cells and total cell numbers under a fluorescent microscope.

## Results

### Butenolide's acute toxicity

The results of acute toxicity tests of butenolide in several marine and freshwater non-target organisms belonging to different taxonomic groups are summarized in Table 4. The lowest L(E)C_50_ was 0.0168 µg ml^−1^ (*Hydroides elegans* larvae) [5] among the freshwater or saltwater representative species of three taxonomic groups of three trophic levels (phytoplankton: *Skeletonema costatum*; crustaceans: *Balanus amphitrite* larvae, *Melita longidactyla*, *Tigriopus japonicus* and *Daphnia magna*; and fish: *Lutjanus erythropterus* and *Danio rerio* embryos) as well as two additional marine taxonomic groups (*Bugula neritina* larvae and *Hydroides elegans* larvae) [5]. According to the technical guidance on risk assessment from the European Chemicals Bureau (http://ecb.jrc.ec.europa.eu/documents/TECHNICAL_GUIDANCE_DOCUMENT/EDITION_2/tgdpart2_2ed.pdf), the AF was set at 1000 in the calculation, resulting in a PNEC of 0.0168 µg l-1. According to guidelines for the testing of chemicals from the Organization for Economic Co-operation and Development (OECD) (http://www.oecd.org/dataoecd/6/14/2483645.pdf), theassessment factor shall be 100 and thus, the PNEC would be 0.168 µg l-1. The positive controls for these toxicity tests are provided in Table S1.

**Table 4:** Effects of butenolide on different organisms.

		EC_10_	EC_50_	LC_10_	LC_50_	Endpoint	Reference
**Non target organisms**							
Micro algae	*Skeletonema costatum*		0.33			5 d IC_50_	
Crustacean	*Melita longidactyla*			2.22	3.02	48 h lethality	
	*Tigriopus japonicus*			1.82	2.56	48 h lethality	
	*Daphnia magna*	0.58	2.34			48 h immobilisation	
Fish	*Lutjanus erythropterus*			0.77	1.32	48 h lethality	
	*Danio rerio*	0.35	0.89	2.75	3.27	Development of pericardial edema/lethality at 55pfh	
**Target organisms (fouling organisms)**							
Crustacean	*Balanus amphitrite*		0.518		>50	48 h settlement	[5]
Bryozoan	*Bugula neritina*		0.199		>50	12 h settlement	[5]
Annelidian	*Hydroides elegans*		0.0168		>2	48 h settlement	[5]

All concentrations are in µg ml^−1^.

### The mechanism of butenolide's acute toxicity on zebrafish embryos

**Figure 2:**
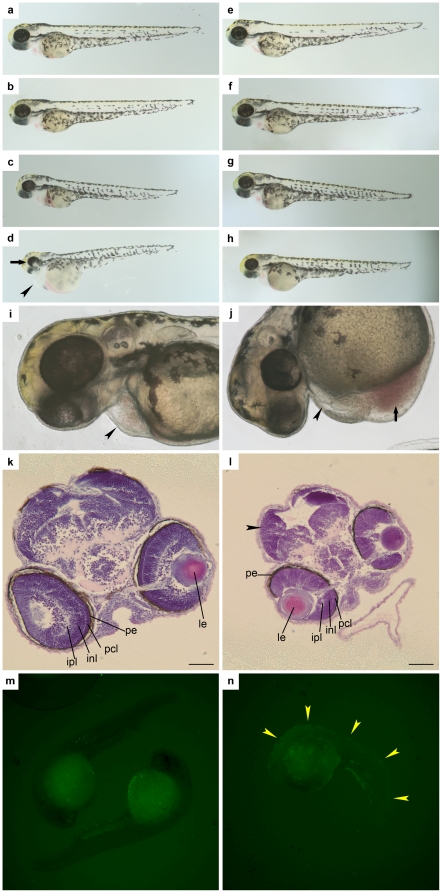
The effect of butenolide on zebrafish embryos. a–d) 55 hpf zebrafish embryos treated with different concentrations of butenolide at the 2-cell stage: a) the control; b) 0.5 µg ml^−1^ butenolide; c) 1.25 µg ml^−1^ butenolide; d) 2.5 µg ml^−1^ butenolide, showing that the treated embryo had smaller eyes (arrow) than the control and pericardial edema (arrowhead). e–h) Zebrafish embryos treated with different concentrations of butenolide at 23 hpf and observed at 55 hpf: e) the control, f) 0.5 µg ml^−1^ butenolide, g) 1.25 µg ml^−1^ butenolide, h) 2.5 µg ml^−1^ butenolide. i–j) High magnification of the pericardial region of zebrafish embryos: i) the control; j) embryo treated with butenolide at 1.25 µg ml^−1^, revealing an enlarged space in the pericardial region (arrowhead) and blood congestion (arrow). k–l) HE staining of the transverse sections through optic nerves of zebrafish embryos 48 hpf (treated at the 2-cell stage): k) control; l) embryo treated with 1.25 µg ml^−1^ butenolide, note that the butenolide-treated zebrafish embryo had a smaller brain (see arrowhead) than the control. le, lens; ipl, inner plexiform layer; inl, inner nuclear layer; pcl, photoreceptor cell layer; pe, pigmented epithelium; bar is 50 µm. m–n) Whole mount TUNEL assay on m) 24 hpf control embryo; n) 24 hpf embryo treated with 1.25 µg ml^−1^ butenolide from the 2 cell stage, revealing the signal on the embryo fish body (arrowhead).

Zebrafish embryos developed pericardial edema (edema around the heart), poor blood circulation, small brains and small eyes within 55 hours of treatment with butenolide (from the 2-cell stage). The severity of these symptoms increased as the concentration of butenolide increased (Table 5, Figure 2a–d, i–l). For example, with a butenolide treatment of 0.5 µg ml-1, the number of embryos that had pericardial edema was only 2 out of 14; but with a treatment of 1.25 µg ml-1, the number increased to 10 out of 12 (Table 5). When the embryos were treated from 23 hours post fertilization (hpf), the phenotype was similar but weaker than those treated from the 2-cell stage (Figure 2e–h). The TUNEL assay results showed an abnormal increase in apoptotic cells in the embryo fish bodies treated with butenolide, indicating that butenolide induced apoptosis in zebrafish embryos (Figure 2m–n). The hatching of butenolide-treated fish was slightly earlier than in the control, but the dose-response relationship was not very clear (Table S2, up to 2.5 µg ml-1). It appears that the butenolide had no effect on the final hatched percentage.

**Table 5:** Effects of different concentrations of butenolide on zebrafish embryos treated at the 2-cell stage and observed at 48 hpf (hours post fertilization).

Concentration of butenolide	Number of zebrafish embryos			
	Total	Normal	Pericardial edema	Died
0 µg ml^−1^	12	12	0	0
0.50 µg ml^−1^	14	12	2	0
1.25 µg ml^−1^	12	2	10	0
2.50 µg ml^−1^	12	0	12	0
5.0 µg ml^−1^	12	0	0	12

### Molecular mechanism of butenolide's acute toxicity in HeLa cells

**Figure 3:**
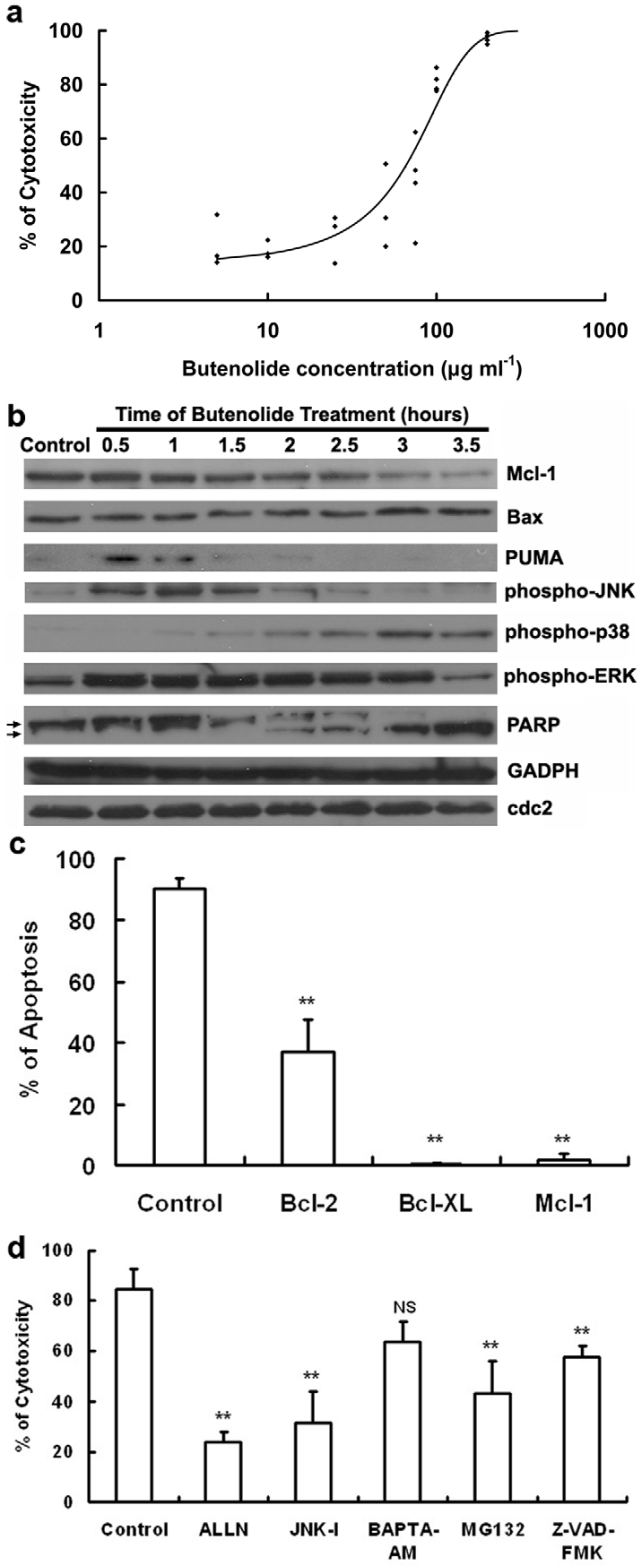
The effect of butenolide on HeLa cells. a) The cytotoxicity of butenolide in HeLa cells (observed at 17 h). The curve was generated according to the logistic regression (R^2^ = 0.897). b) Effect of butenolide on several apoptosis-related proteins in HeLa cells. GADPH and cdc-2 served as the internal loading controls. c) Over-expression of Bcl-2 family members protected-HeLa cells from butenolide-induced apoptosis at different degrees. Note that the PARP is cleaved after 2 h of butenolide treatment. d) Effect of inhibitors on butenolide-treated HeLa cells (see text). JNK-I: JNK inhibitors. ** indicates significant difference between the treatment and the control (100 µg ml^−1^ butenolide) at p<0.01. NS indicates no significant difference between the treatment and the control. Error bars represent standard deviations.

The concentration of butenolide that led to toxicity in HeLa, Ptk2, Sf9, HL-60 and K562 cells was similar, whereas the primary neuron cells were more sensitive to butenolide treatment than the other cell types (Table S3). Because of their ease of manipulation and the consistency and robustness of their results, HeLa cells were used for the subsequent toxicology studies (Figure 3a). Similar to its effect on zebrafish embryos, butenolide also induced apoptosis in HeLa cells, which was confirmed by counter staining with Hoechst 33342 (data not shown).

As revealed by the Western blot analysis (Figure 3b), the caspase substrate protein PARP (poly ADP ribose polymerase) showed cleavage after 2 h of treatment, suggesting the activation of protease caspase-3 and execution of apoptosis.

In the early stages (within 2 h) of treatment with butenolide, MAPKs family members JNK (c-Jun N-terminal kinases) and ERK (extracellular signal-regulated kinases) were activated (phosphorylated), whereas p38 was activated later (after PARP cleavage) (Figure 3b). The JNK inhibitor SP600125 partially inhibited butenolide-induced HeLa cell apoptosis (Figure 3d), whereas p38 and ERK inhibitors had no significant inhibiting effect (data not shown), suggesting that JNK is involved in butenolide-induced apoptosis and is the most important MAPK family protein involved in this process.

The expression level of the Bcl-2 family protein Bax did not change with butenolide treatment (Figure 3b). However, the Bcl-2 family proteins PUMA and Mcl-1 were up-regulated in the early stages of butenolide treatment (Figure 3b), suggesting that Bcl-2 family proteins are involved in butenolide-induced apoptosis. The over-expression of the anti-apoptotic Bcl-2 family proteins, Bcl-2, Bcl-XL and Mcl-1, protected the cells from butenolide-induced apoptosis (Figure 3c), indicating that butenolide's pro-apoptotic effect involves Bcl-2 family proteins.

The pan-caspase inhibitor z-VAD-fmk [12], [13] partially inhibited butenolide-induced apoptosis in the HeLa cells (Figure 3d), indicating that butenolide-induced apoptosis in HeLa cells requires caspases. The proteasome, lysosome and calpain inhibitors ALLN and MG132 [14], [15] also partially protected the cells from butenolide-induced apoptosis (Figure 3d). However, calpain is a calcium-activated protease [16], and the selective intracellular Ca2+ stores chelator BAPTA-AM [17], [18] had no significant rescuing effect (Figure 3d), indicating that calpain might have less effect on butenolide-induced apoptosis, and that proteasomes and/or lysosomes are involved in butenolide-induced apoptosis.

## Discussion

To assess the toxicity of a new antifouling compound, we calculated the predicted no effect concentration (PNEC) according to two standards with different AFs. When calculated and compared using the OECD's standard, the PNEC of butenolide is among the highest in representative alternative new biocides [4], [19].

Regarding the selection of test species, we investigated more organisms than the minimum requirements, which requires three species from three trophic levels, and preferably two additional marine taxonomic groups for PNEC calculations [4], [6]. Specifically, we considered several fouling species in the PNEC calculation in addition to non-target organisms. Although fouling organisms are target organisms for antifouling compounds, their ordinary lives should not be affected by these compounds where these organisms were harmless; and the effective concentrations on these species are important references for the prediction of the effective concentrations in other species. As of now, the fouling species *H. elegans*, *B. neritina*, and *B. amphitrite* were found to be among the most sensitive species to butenolide. Species-selectivity ratios/indices were used to assess the species-selectivity of herbicides [20], [21]. Here, we used a similar method to compare the species-selectivity of several antifouling compounds (Table 6) by the following equation:
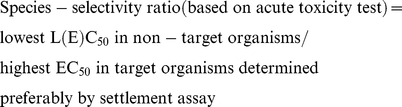
This ratio indicates the compound's species-specificity under the most extreme conditions, which are most likely to exist in places near the antifouling coatings. A higher value suggests higher specificity towards target organisms. The species-selectivity ratio for butenolide is 0.635, which is quite high compared to other biocides (Table 6), suggesting that butenolide is comparatively more specific towards fouling organisms than are other biocides. This makes butenolide desirable as an antifouling compound.

**Table 6:** Species-selectivity ratio of biocides^1^.

Biocide	Species	Highest EC_50_ in target organism	Lowest L(E)C_50_ in non-target organism	Endpoint	Species-selectivity ratio	Reference
Butenolide	*Balanus amphitrite* (cyprid)	0.518		48 h settlement rate	0.64	[5]
	*Skeletonema costatum*		0.33	5 d I(E)C_50_		This work
Chlorothalonil	*Hydroides elegans* (trochophore)	0.012		48 h LC_50_	0.37	[38]
	*Thalassiosira pseudonana*		0.0044	96 h EC_50_		[38]
TBT	*Balanus amphitrite* (cyprid)	0.034		24 h LC_50_	0.032	[38]
	*Skeletonema costatum*		0.0011	96 h EC_50_		[38]
CuPT	*Balanus amphitrite* (cyprid)	0.063		24 h LC_50_	0.011	[38]
	*Thalassiosira pseudonana*		0.0007	96 h EC_50_		[38]
ZnPT	*Balanus amphitrite* (larvae)	0.21		24 h LC_50_	0.0024	[38]
	*Thalassiosira pseudonana*		0.0005	96 h EC_50_		[38]
Seanine 211	*Bugula neritina* (swimming larvae)	2.5		48 h settlement EC_50_	0.00108	Unpublished data
	*Oncorhynchus mykiss*		0.0027	72 h IC_50_		[4]
Diuron	*Balanus amphitrite* (larvae)	21		24 h LC_50_	0.0002	[38]
	*Thalassiosira pseudonana*		0.0043	96 h EC_50_		[38]
Irgarol	*Hydroides elegans* (trochophore)	2.6		48 h LC_50_	0.00015	[38]
	*Thalassiosira pseudonana*		0.0004	96 h EC_50_		[38]

All concentrations are in µg ml^−1^.

1 The species-selectivity ratios for antifouling compounds other than butenolide are tentative estimations from limited sources.

The species-selectivity ratio of butenolide is much lower than its pharmaceutical ratios. The pharmaceutical ratios (LC_50_/EC_50_) were based on single species. When used on fouling organisms, the pharmaceutical ratio assessed the specificity of the compound's effect, with a higher value suggesting higher specificity toward the fouling process and lower off-target side effects. The pharmaceutical ratio of butenolide was quite high in target organisms (>97 for *B. amphitrite*, >250 for *B. neritina*, >119 for *H. elegans*) [5], but much lower in the embryos of the non-target organism *D. rerio* (3.67). The exact mechanism for this huge difference is unclear, but one possibility is that butenolide has a very specific effect on the fouling process in fouling organisms but much less specific effects on non-target organisms. The variation in the L(E)C_50_ of butenolide in different non-target species is within an order of magnitude, which is quite low among new antifouling biocides [4], suggesting that butenolide does not discriminate much among non-target organisms and is less likely to cause selective loss of a particular non-target organism in the environment. The L(E)C_50_ varies by at least an order of magnitude between target organisms; and LC_50_ of non-target crustaceans was much lower than that of the crustacean *B. Amphitrite*. The pharmacology of butenolide in different species should be investigated to clarify the reasons for these inter-species differences.

To guarantee the safe usage of butenolide as antifouling compound, its degradation should be further studied to ensure that the effective concentrations are reached only within a very limited space near the coating. Our preliminary data on butenolide degradation showed that it can be easily degraded after its release into natural sea water (unpublished results).

During zebrafish organogenesis, the heart is the first organ formed in the developing embryo, and the development of other organs depends on blood circulation in the embryo. Pericardial edema was the first symptom observed after treatment with butenolide, and blood circulation in the embryos was in turn affected. It is possible that the developmental defects/retardation in the brains and eyes were caused by insufficient blood circulation. We therefore focused only on pericardial edema and looked for similar phenotypes. Using the key words “pericardial edema” to search the zebrafish embryo developmental defect database, we found that the symptoms caused by butenolide were similar to the phenotype found in pro-caspase-3 over-expression mutants [22], which have pericardial edema, small brains, small eyes and abnormal increases of apoptotic cells in the body. The similarity indicates that butenolide induces apoptosis in zebrafish embryos and suggests that butenolide's phenotype in zebrafish embryos was the result of apoptosis. Therefore, pro-apoptosis is likely to be the major effect of butenolide on this organism.

However, there were some differences between the pro-caspase-3 over-expression mutants and the butenolide-treated embryos. In the pro-caspase-3 mutants, some retina cells were lost in the eyes, especially in the photoreceptor cell layer, and the number of apoptotic cells in the retina increased [22]. However, in the butenolide-treated embryos, the eyes were well structured; the retina cells were well organized and densely compacted; and there was no obvious cell loss or specific sensitivity of the photoreceptor cell layer to butenolide. Also, we did not observe any increase in the number of apoptotic cells in the retina in our TUNEL assays of both whole mount and tissue sections (data not shown). Perhaps the cause of the small eye phenotype differs between the pro-caspase-3 over-expression mutants and the butenolide-treated embryos. The higher activity of caspase-3 in the retina might lead to retina cell loss and small-eye defects in the pro-caspase-3 overexpression mutants, whereas pericardial edema and poor blood circulation might be the reasons for the defects/developmental retardation in the eyes of the butenolide-treated embryos. These results suggest that the mechanism of apoptosis induced by butenolide involves more than just the overall higher activity of caspase-3.

To study the molecular mechanism of butenolide-induced apoptosis, we used a representative cell culture to examine the mechanism of butenolide's direct effect. The LC_50_s in the tested cell lines were higher than in the non-target organisms. Although the reason for this discrepancy is unclear, it is important to understand that the direct effect on cells differs from the most prominent effects on the whole organism, and that the host species, cell type and culture method could all influence the sensitivity of the cell to butenolide. Among several cell types, HeLa cells were chosen for molecular toxicology study, because they are very consistent, robust, and easy to manipulate, and butenolide's effective concentration in this cell line was similar to that in most of the other tested cell lines, including the insect cell line Sf9. The concentration of butenolide for the molecular mechanism study was 100 µg ml^−1^. This concentration was just high enough to cause sufficient cytotoxicity (Figure 3a), while low enough to avoid necrosis as confirmed by counter staining with Hoechst 33342 (data not shown).

The MAPKs family includes ERK1/2, JNK/SAPK, p38 and ERK5. These proteins are involved in the survival, proliferation and differentiation of cells [23]. Bcl-2 family proteins exhibit either pro- or anti-apoptotic activities and regulate the mitochondrial pathways of apoptosis by controlling the permeabilization of the outer mitochondrial membrane [24]. We found that butenolide-induced apoptosis in HeLa cells involved JNK and Bcl-2 family proteins. This finding was supported by our inhibitor assays and over-expression assays, respectively. For instance, the JNK inhibitors inhibited butenolide-induced apoptosis, whereas the p38 and ERK inhibitors did not have significant inhibiting effects. JNK was activated early, suggesting that its regulatory role may be in the early stages. The Bax protein, which forms the Mitochondrial Outer Membrane Permeabilization Pore (MOMPP) and induces apoptosis [25], [26], did not change in expression level (Figure 3b) at the early stage (within 2 h); whereas PUMA, which initiates apoptosis by dissociating Bax and Bcl-XL, thereby promoting Bax multimerization and mitochondrial translocation [27], was activated in the early stages of butenolide treatment (Figure 3b). This evidence suggests that Bax is activated early by PUMA upon butenolide treatment. Furthermore, Mcl-1, which prevents apoptosis by inhibiting MOMPP formation [28], decreased in expression level after 1.5 h (Figure 3b), which is later than PUMA's response, and earlier than the cleavage of PARP, suggesting that Mcl-1 is also involved in the early regulation of butenolide-induced apoptosis. The over-expression of Bcl-2 family members that inhibit MOMPP formation (Bcl-2, Bcl-XL or Mcl-1) [25], [28] protected cells from butenolide-induced apoptosis (Figure 3c), indicating that Bcl-2 family members mediate butenolide-induced apoptosis.

Proteases are executioners of apoptosis. After apoptosis begins, cellular organelles undergo organized degradation by activated proteases [29], [30]. Proteases also play a regulatory role in apoptosis. For example, proteases in proteasome regulate the degradation of some endogenous Inhibitors of Apoptosis (IAP) [31]. If the proteasome activity is inhibited, the degradation of some IAP would be subsequently inhibited, leaving more IAP available to inhibit apoptosis. In this study, both pan-caspase inhibitor and proteasome/lysosome inhibitors partially protected HeLa cells from butenolide-induced apoptosis (Figure 3d), indicating the necessity of these proteases in butenolide-induced apoptosis. Whether caspase and proteasome/lysosome were involved before the execution of butenolide-induced apoptosis remains unclear.

Besides apoptosis, we also tested the effect of butenolide on several other cell signaling pathways. Butenolide treatment did not change the proportion of interphase cells (up to 50 µg l^−1^), which was verified by FACS analysis (data not shown); 10 µM purvalanol A (a CDK1 inhibitor) did not inhibit butenolide-induced apoptosis (data not shown), suggesting that butenolide may have less effect on the cell cycle.

In the cyprid larvae of the marine fouling organism *Balanus amphitrite*, caspase-3 activity decreases 24 h after molting, but it is partially sustained by butenolide treatment [32]. However, butenolide can inhibit the positive phototactic behavior of *B. amphitrite* cyprids within a few minutes after treatment [33], which is unlikely to be due to a pro-apoptotic effect, suggesting that butenolide has other effects on *B. amphitrite* than only pro-apoptotic activity. The relationship between the pro-apoptotic effect and the antifouling effect of butenolide remains unknown. Another type of butenolide [2-(6-hydroxy-6-methyl-octyl)-2H-furan-5-one], which is structurally similar to the butenolide studied here, exhibited novel anti-parasitic activities specifically against *Trypanosoma brucei brucei* [34]. It is possible that anti-parasitic butenolide also has pro-apoptotic effects.

In summary, the antifouling compound butenolide's PNEC was 0.016 µg l^−1^ according to the European Chemicals Bureau, and 0.16 µg l^−1^ according to the Organization for Economic Co-operation and Development (OECD). As of now, the fouling species *Hydroides elegans* was found to be the most sensitive species to butenolide treatment and it could be chosen for chronic toxicity tests. Mechanistically, butenolide induced apoptosis in both zebrafish embryos and HeLa cells. JNK activation, Bcl-2 family members, caspases and proteasome/lysosome activation were involved in butenolide-induced apoptosis in HeLa cell lines. The results reported here increased our understanding on butenolide's toxicity and toxicology. For risk assessment, the degradation, bioaccumulation, and the predicted environment concentration (PEC) of butenolide should be further measured and calculated [6] in the future.

## Supporting Information

Table S1 **Positive controls for toxicity tests.** All concentration units are µg ml^−1^.
(DOC)

Table S2 **The effect of butenolide on zebrafish hatching.**
(DOC)

Table S3 **The toxicity of butenolide in several cell lines.** All concentration units are µg ml^−1^.
(DOC)
