# Supplementary material for: Acute Toxicity of the Antifouling Compound Butenolide in Non-Target Organisms
Source: PLoS One. 2011 Aug 29;6(8):e23803. doi: 10.1371/journal.pone.0023803 (PMC3163639; doi:10.1371/journal.pone.0023803)
Supplement: Table S1 — Positive controls for toxicity tests. All concentration units are µg ml−1. (DOC) [file pone.0023803.s001.doc]

Table S1. Positive controls for toxicity tests. All concentration units are µg ml^-1^.

|  | Chemical | EC_50_ | LC_50_ | Endpoint |
| --- | --- | --- | --- | --- |
| *Skeletonema costatum* | Cd^2+^ |  | 0.131 | 5d IC_50_ |
| *Melita longidactyla* | Cd^2+^ |  | 1.26 | 48h lethality |
| *Tigriopus japonicus* | Cd^2+^ |  | 4.0 | 48h lethality |
| *Daphnia magna* | Cd^2+^ | 0.0173 |  | 48h immobilization |
| *Lutjanus erythropterus* | Cd^2+^ |  | 14.7 | 48h lethality |
