# Supplementary material for: Acute Toxicity of the Antifouling Compound Butenolide in Non-Target Organisms
Source: PLoS One. 2011 Aug 29;6(8):e23803. doi: 10.1371/journal.pone.0023803 (PMC3163639; doi:10.1371/journal.pone.0023803)
Supplement: Table S2 — The effect of butenolide on zebrafish hatching. (DOC) [file pone.0023803.s002.doc]

Table S2. The effect of butenolide on zebrafish hatching.

| Butenolide conc. (µg ml^-1^) | Total | Hatched before 58h | Hatched before 70h |
| --- | --- | --- | --- |
| 0 | 16 | 1 | 3 |
| 0.5 | 17 | 3 | 8 |
| 1.0 | 15 | 2 | 7 |
| 1.5 | 18 | 1 | 8 |
| 2.0 | 13 | 4 | 8 |
| 2.5 | 11 | 3 | 5 |
