# Supplementary material for: Acute Toxicity of the Antifouling Compound Butenolide in Non-Target Organisms
Source: PLoS One. 2011 Aug 29;6(8):e23803. doi: 10.1371/journal.pone.0023803 (PMC3163639; doi:10.1371/journal.pone.0023803)
Supplement: Table S3 — The toxicity of butenolide in several cell lines. All concentration units are µg ml−1. (DOC) [file pone.0023803.s003.doc]

Table S3. The toxicity of butenolide in several cell lines. All concentration units are µg ml^-1^.

|  | LC_10_ | LC_50_ | Endpoint |
| --- | --- | --- | --- |
| HeLa cell | 29 | 68 | 17h cytotoxicity |
| Ptk2 cell | 20 | 61 | 12h cytotoxicity |
| Sf9 cell | 27 | 59 | 24h cytotoxicity |
| HL-60 cell | 19 | 54 | 24h cytotoxicity |
| K562 cell | 27 | 53 | 24h cytotoxicity |
| Primary cortical neuron | 12 | 18 | 48h cytotoxicity |
